# Utilization of complementary and alternative medicine (CAM) among children from a German birth cohort (GINIplus): patterns, costs, and trends of use

**DOI:** 10.1186/s12906-015-0569-8

**Published:** 2015-03-10

**Authors:** Salvatore Italia, Helmut Brand, Joachim Heinrich, Dietrich Berdel, Andrea von Berg, Silke Britta Wolfenstetter

**Affiliations:** Department of International Health, School for Public Health and Primary Care (CAPHRI), Faculty of Health, Medicine and Life Sciences, Maastricht University, Maastricht, The Netherlands; Helmholtz Zentrum München, German Research Center for Environmental Health, Institute of Health Economics and Health Care Management, Neuherberg, Germany; Helmholtz Zentrum München, German Research Center for Environmental Health, Institute of Epidemiology I, Neuherberg, Germany; Marien-Hospital Wesel, Department of Pediatrics – Research Institute, Wesel, Germany

**Keywords:** Complementary therapies, CAM, Homeopathy, Phytotherapy, Child, Germany, Drug utilization, Socioeconomic factors, Trends

## Abstract

**Background:**

The use of complementary and alternative medicine (CAM) is widespread among children in Germany and other European countries. Only a few studies are available on trends in pediatric CAM use over time. The study’s objective was to present updated results for prevalence, predictors, and costs of CAM use among German children and a comparison with findings from a previous follow-up of the same birth cohort.

**Methods:**

Data were collected for 3013 children on their utilization of medicinal products (during the last 4 weeks) and consultation with CAM providers (in the preceding year) from a German birth cohort study (GINIplus, 15-year follow-up) using a self-administered questionnaire. The reported medicinal CAMs were classified into six categories (homeopathy, herbal drugs, nutritionals, minerals and trace elements, microorganisms, further CAM). Drug prices were traced using pharmaceutical identification numbers (PZNs), or otherwise conservatively estimated. Finally, the results were compared with data obtained from the 10-year follow-up of the same birth cohort study by adopting the identical methodology.

**Results:**

In all, 26% of the reported 2489 drugs were medicinal CAM. The 4-week prevalence for homeopathy and herbal drug use was 7.5% and 5.6%, respectively. Some 13.9% of the children used at least one type of medicinal CAM in the preceding 4 weeks. The 1-year prevalence for consultation with CAM providers was 10.8%. From the drugs identified as CAM, 53.7% were homeopathic remedies, and 30.8% were herbal drugs.

Factors associated with higher medicinal CAM use were female gender, residing in Munich, and higher maternal education.

A homeopathy user utilized on average homeopathic remedies worth EUR 15.28. The corresponding figure for herbal drug users was EUR 16.02, and EUR 18.72 for overall medicinal CAM users.

Compared with the 10-year follow-up, the prevalence of homeopathy use was more than halved (−52%) and dropped substantially for herbal drug use (−36%) and overall CAM use (−38%) as well.

**Conclusion:**

CAM use among 15-year-old children in the GINIplus cohort is popular, but decreased noticeably compared with children from the same cohort at the age of 10 years. This is possibly mainly because German health legislation normally covers CAM for children younger than 12 years only.

## Background

Therapy approaches that are not part of conventional medicine are often referred to as complementary and alternative medicine (CAM), although there is no universally valid definition of CAM. However, there seems to be an unbroken high demand for such kinds of therapy approaches in the European population and outside Europe as well. Some recently published reviews give updated overviews on the prevalence of CAM use in adults [1,2] and children [3,4]. Owing to different methodology and CAM definition, the findings of the included studies on CAM use vary widely with respect to the prevalence and predictors of use. For instance, overall CAM use in children without chronic conditions was reported to be between 1.8% and 87.6%, depending on included CAM modalities, country, and underlying recall period [3].

When looking at specific CAM categories, many of the reviewed studies listed homeopathy and herbal drugs among the most popular types of CAM. Within Europe, Germany ranks between the countries with the highest prevalence rates for homeopathy and herbal drug use in children. For homeopathy, prevalence rates of 27.7% (1-year prevalence) [5] and 14.3% (4-week prevalence) [6] were reported in German children. Other European countries with high prevalence rates for homeopathy use are The Netherlands with 14.6% (1-year prevalence) [7] and the UK with 16.9% (lifetime use) [8]. With concern to herbal drugs, a 2010 publication found 85.5% of German children [9] using herbal drugs (lifetime use). In Turkey, the prevalence of pediatric use of herbal drugs was 58.6% (1-year prevalence) [10]. The evidently high popularity of non-conventional medicine in Germany and other countries makes CAM use a relevant public health topic.

This article presents data from the recently completed 15-year follow-up of a German birth cohort study. The aim was to extract prevalence rates and predictors for the utilization of various CAM modalities (homeopathy use, herbal drug use, medicinal CAM utilization in general, and consultation with CAM providers). Furthermore, expenditures on pediatric CAM use were analyzed. Finally, in order to detect possible time trends and differences in CAM use, the results were compared with the findings from the 10-year follow-up of a smaller but similarly composed sample from the same birth cohort.

## Methods

### Study population

GINIplus (German Infant study on the Influence of Nutrition Intervention plus environmental and genetic influences on allergy development) is a German birth cohort study [11]. It started with 5991 healthy full-term newborns (children with a birth weight <2500 g were not eligible for inclusion), who were recruited between September 1995 and June 1998 from obstetric clinics in an urban region of southern Germany (Munich) and a more rural region in the western part of Germany (Wesel).

For the 15-year follow-up, 3895 participants were contacted between January 2011 and September 2013. With regard to the season, 27% of the questionnaires were collected in winter (January–March), 29% in spring (April–June), 26% in summer (July–September), and the remaining 18% in autumn (October–December). Among other things, the main questionnaire assessed the children’s gender, parental income and education, and consultation with various alternative health care providers during the previous 12 months.

In addition to the main questionnaire, a self-adminis-tered questionnaire on the consumption of drugs and medicinal products was included based on an almost identical questionnaire that had already been adopted for the 10-year follow-up. The design of the questionnaire on drug utilization corresponds to the validated questionnaire from the German KiGGS-Study that was conducted with 17641 children [12]. Parents/legal guardians were invited to report the drugs their child used during the last 4 weeks by entering the drugs’ names into designated spaces or attaching the empty drug packages to the questionnaire.

The exact number of drugs used was assessed by an additional question in case the limited number (five) of designated spaces would not suffice to report all drugs utilized.

Moreover, the participants were also asked to enter the pharmaceutical identification number (PZN) of the reported drugs. The PZN, which is printed on the drug package, precisely identifies the drug utilized and provides further information such as the size of the package, the dosage, the pharmaceutical manufacturer, the listed price, etc.

To avoid ambiguity of interpretation, the authors would like to note that we also considered preparations that are no medicinal products in the strict sense (e.g., nutritional supplements), but were reported as drugs utilized by the participating children.

### Drug classification

All reported drugs were classified into several therapeutic categories. The following medicinal CAM modalities were defined and extracted from the entity of reported drugs:

1. Homeopathic/anthroposophic drugs (afterwards referred to as ‘Homeopathy’): Drugs that have been prepared according to the production specification of the Homeopathic Pharmacopoeia HAB [13], including anthroposophic remedies and biochemic remedies (Schuessler salts).
2. Herbal drugs: Herbal extracts and their preparations, teas. Preparations containing active pharmaceutical ingredients of herbal origin (e.g., codeine) that are available by prescription only were excluded.
3. Nutritionals: Vitamins and combined food supplements. Preparations containing vitamin D for prophylaxis according to medical guidelines were excluded.
4. Minerals and trace elements: Mono-preparations of minerals or trace elements such as calcium, magnesium, selenium, etc. Preparations containing iodide and/or fluoride for prophylaxis according to medical guidelines were excluded.
5. Microorganisms: Non-pathogenic microorganisms or their metabolites used to regulate the intestinal flora or stimulate the immune system.
6. Further medicinal CAM: Bach flower, traditional Chinese medicine, etc.

As well as medicinal CAM use, consultation with CAM providers was assessed during the previous 12 months (non-medical health provider (‘Heilpraktiker’), homeopath, osteopath, and ‘others’) for the child’s disease or disorder.

### Cost accounting

Parents were asked to report expenditures for consultations with CAM providers. Prices for medicinal CAM were traced via PZNs (official pharmacy prices from the ‘Lauer’ price list as of August 2012). For drug entries without PZNs, conservative assumptions were made (e.g., smallest package size, most favorable price).

### Comparison of the results

The results were compared with data based on the 10-year follow-up of the combined GINIplus and LISAplus birth cohort studies (n = 3642) [6]. For comparison, only data from the GINIplus subset were used, which included 2065 children from Munich and Wesel. The distribution of the participants with regard to gender, study area, maternal educational background, and parental income background was very similar to the composition of the GINIplus cohort of the 15-year follow-up (n = 3013). Furthermore, the same methodology (drug classification, logistic regression model, etc.) was adopted for the analysis of both follow-ups.

### Outcome definition and statistical analysis

Several outcomes were defined for the statistical analysis.

Those participants who reported utilization of at least one homeopathic drug during the past 4 weeks were defined as ‘homeopathy users’ and those taking at least one herbal drug as ‘herbal drug users’, respectively. ‘Overall CAM users’ took at least one drug from the therapeutic categories 1–6. Finally, a ‘CAM provider user’ consulted at least once during the past year with a non-medical health provider (‘Heilpraktiker’), a homeopath, an osteopath, or another type of CAM provider.

The statistical analysis was performed with the SAS software package (SAS Institute Inc., Cary, NC, USA, version 9.3). Odds ratios (ORs) and their 95% confidence intervals (CI) were obtained using a multivariate logistic regression model. The significance level for the estimates was set at p < 0.05. All independent variables included in the model were checked using the F-test for significance. Bivariate associations between the independent variables and users’ prevalence rates were analyzed by Chi^2^ test (p < 0.05).

To define educational status, the mothers’ educational background was classified into four levels based on their highest school degree:

- Level 1: secondary school
- Level 2: junior high school
- Level 3: baccalaureate (= qualification for university entrance)
- Level 4: university degree

Mothers who reported no school degree at all (n = 5) were allocated to education level 1. Entries for mothers (n = 4) reporting another (not further specified) kind of school degree than those listed above, were treated as missing values for educational background.

The income status was defined using the median equivalence income (MEI) for 2012 (€1633 net/month) [14] where the household members were weighted according to the new scale of the Organisation for Economic Co-operation and Development (OECD) [15]. The income cut-offs were chosen according to the definition of poverty (60% of MEI) [16].

The GINIplus cohort obtained approval from the ethics committee of the Bavarian Medical Council and the Medical Council of North Rhine-Westphalia. Furthermore, written informed consent was given by the participants’ parents or legal guardians and by participants.

## Results

### Cohort structure and prevalence of CAM use

Out of 3895 distributed questionnaires assessing drug utilization, 3013 were completed and returned, yielding a response rate of 77.4%. The children’s average age was 15.1 years, ranging between 14.5 years and 16.8 years. Mothers completed 85.5% of the questionnaires, fathers 5.1%, and questionnaires completed by both parents accounted for 2.7% (missing values = 6.7%). Compared with the baseline survey, the parents of those children who participated in the 15-year follow-up have higher levels of school education and income. Table 1 shows the cohort structure and the stratified prevalence rates of CAM use. The 4-week prevalence (95% CI) of homeopathy use was 7.5% [(6.5;8.5) n = 226], whereas 170 children [5.6% (4.8;6.5)] used herbal drugs. As defined in the methods section, 10 prescription drugs containing opium alkaloids (noscapine, morphine) or allergens extracted from pollen were excluded from the CAM modality ‘herbal drugs’. Looking at all CAM categories, 418 children [13.9% (12.6;15.1)] used at least one drug from the CAM categories 1–6.

**Table 1:** **Characteristics of the GINIplus cohort and prevalence of use**

		**Prevalence of use in %**			
	**n**	**Homeopathy** ^**1**^	**Herbal drugs** ^**1**^	**All medicinal CAM** ^**1**^	**CAM providers** ^**2**^
**Gender**					
Male	1500	6.1	4.8	11.5	9.9
Female	1513	8.9	6.5	16.3	11.6
**Study area**					
Munich	1457	9.0	5.8	15.7	14.0
Wesel	1556	6.1	5.5	12.2	7.7
**Maternal education**					
Secondary school	380	5.0	3.2	9.0	4.2
Junior high school	1251	7.7	5.6	14.2	10.4
Baccalaureate	580	8.6	6.7	15.3	12.4
University degree	795	7.7	6.2	14.6	13.2
**Household income**					
≤60% of MEI	529	6.8	4.7	11.2	6.6
60–100% of MEI	985	7.7	6.3	15.2	10.5
>100% of MEI	1071	7.8	5.6	14.4	13.4
Total	3013	7.5	5.6	13.9	10.8

Owing to missing values, the strata may not add up to the total number of participants.

MEI = median equivalence income.

^1^Use within the last 4 weeks.

^2^Consultation with any type of CAM provider in the previous 12 months.

CAM provider = non-medical health practitioner, homeopath, osteopath, and ‘others’.

**Figure 1:**
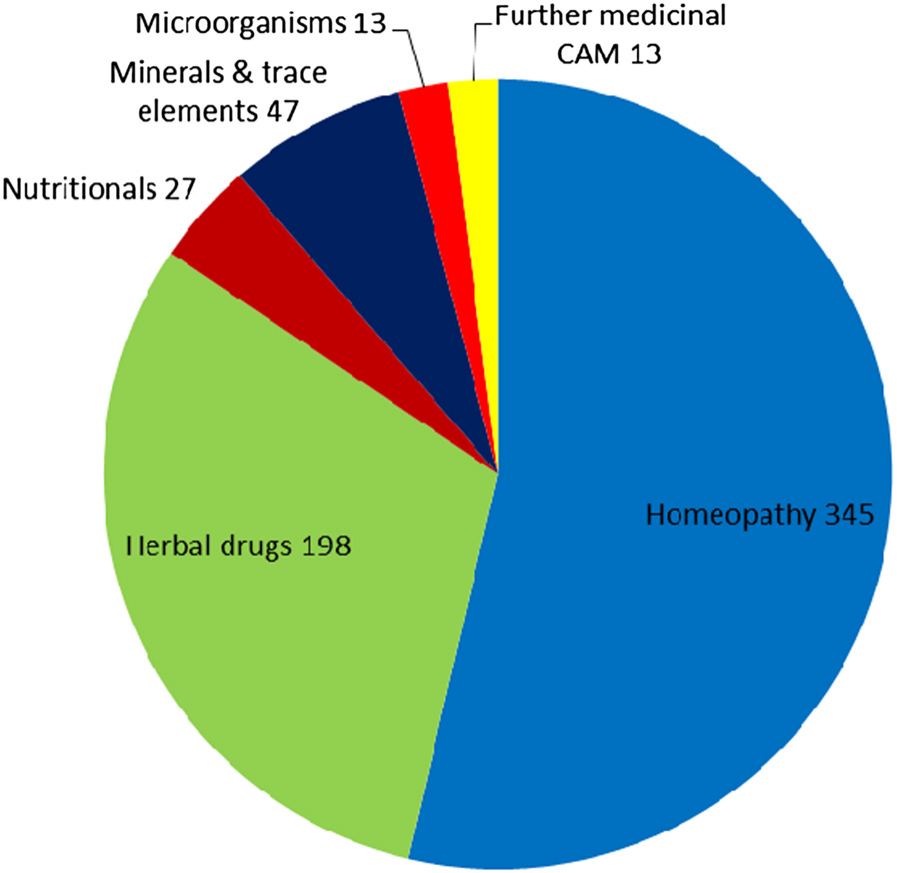
**Proportion of the single medicinal CAM modalities in all reported medicinal CAM (n = 643).**

In sum, 1234 of the 3013 participating children reported having used at least one drug during the 4 weeks prior to the assessment date. The total number of drugs utilized was 2489, of which 2444 could be allocated to a therapeutic category. The remaining 45 drug entries did not provide enough information to identify the therapeutic category and were therefore interpreted as drug use only. The majority of utilized drugs were conventional drugs with chemically active pharmaceutical ingredients such as ibuprofen or paracetamol. Nevertheless, about 26% (n = 643) belonged to the non-conventional drug categories, as defined above in the section ‘drug classification’. Of the 643 identified CAM, 642 were available without medical prescription. The detailed distribution of non-conventional drugs over the various CAM modalities is shown in Figure 1. Homeopathic remedies were the most commonly used CAM modality (14.1% of all identified drugs), followed by herbal drugs (8.1%), minerals and trace elements (1.9%), and nutritionals (1.1%). Other medicinal CAM modalities such as Bach flower remedies or traditional Chinese medicine played only a marginal role.

The most mentioned homeopathic and herbal drugs are displayed with their ATC codes in Table 2. Concerning homeopathy, 141 of the 345 reported homeopathic remedies were combined preparations. A further 146 were single homeopathic drugs such as Arnica (n = 26), Belladonna (n = 9), and Gelsemium (n = 9), which were the most frequent single homeopathic remedies. Twelve drugs were clearly identified as anthroposophic remedies. Single remedies as well as biochemic remedies according to Dr. Schuessler are partly used for several disease patterns. An allocation to ATC categories is therefore not possible.

**Table 2:** **Most frequent homeopathic and herbal drugs**

**Homeopathic drugs (n = 345)**				**Herbal drugs (n = 198)**			
**ATC code**	**Remedy**	**n**	**(in %)**	**ATC code**	**Remedy**	**n**	**(in %)**
R05XH20	Flu remedies*	31	(9.1)	R01BP30	Systemic rhinologicals*	50	(25.3)
R02AH20	Therapeutics for throat and pharynx*	14	(4.1)	R05CA19	Expectorants (Myrtol® standardized)	23	(11.6)
R01BH20	Rhinologicals*	9	(2.6)	R05CP02	Ivy leaves	22	(11.1)
S02DH20	Otologicals*	8	(2.3)	A03FP30	Prokinetics*	18	(9.1)
M02AH20	Remedies for muscle and joint pains*	7	(2.0)	R05CP05	Pelargonium root	18	(9.1)
/	Single preparations	146	(42.3)	G02CP01	Vitex agnus-castus	10	(5.1)
/	Biochemic remedies (= Schuessler salts)	58	(16.8)	R05CA25	Expectorants (1,8-Cineol)	6	(3.0)

ATC = anatomical therapeutical classification.

*Combined preparations.

With respect to herbal drugs, more than 70% of the 198 drugs from this category were used for the treatment of coughs and colds. The mean duration of overall CAM use was 11.1 days (median = 6). The corresponding figures for homeopathy and herbal drugs were 9.7 days (median = 5) and 8.6 days (median = 5). In comparison with CAM, the mean duration of conventional drug use was 11.4 days (median = 5).

About 47% of the minerals and trace elements (n = 47) were preparations containing iron (n = 22). Medicinal mono-preparations with iodide (n = 43), fluoride (n = 1), and vitamin D (n = 6) were not defined as CAM, as they are normally used for prophylaxis according to medical guidelines.

The prevalence for consultation with CAM providers (within the previous 12 months) was lower than for medicinal CAM use. Some 144 children (4.8%) visited a non-medical health provider (‘Heilpraktiker’). Consultation with a homeopath was reported for 98 children (3.3%), with an osteopath for 102 children (3.4%), and 38 participants (1.3%) consulted with other CAM providers. Overall, 324 children [10.8% (9.6;11.9)] visited at least one type of CAM provider during the 12 months prior to the assessment date.

### Predictors of CAM use

Table 3 summarizes the ORs for predicting factors for CAM use.

**Table 3:** **Predictors of complementary and alternative medicine use**

	**Adjusted odds ratio of utilization (and 95% confidence interval)**							
	**Homeopathy**		**Herbal drugs**		**All medicinal CAM**		**CAM providers**	
**Gender**								
Male	Reference		Reference		Reference		Reference	
Female	1.48*	(1.12–1.95)	1.36	(0.99–1.86)	1.49*	(1.21–1.84)	1.19	(0.94–1.50)
**Study area**								
Munich	Reference		Reference		Reference		Reference	
Wesel	0.62*	(0.46–0.84)	0.97	(0.69–1.36)	**0.75**	(0.59–0.94)	0.57**	(0.44–0.74)
**Maternal education**								
Secondary school	Reference		Reference		Reference		Reference	
Junior high school	1.61	(0.96–2.68)	1.79	(0.96–3.36)	**1.66**	(1.12–2.45)	2.57*	(1.50–4.39)
Baccalaureate	1.72	(0.99–3.01)	**2.15**	(1.10–4.21)	**1.71**	(1.12–2.62)	2.80*	(1.59–4.92)
University degree	1.38	(0.79–2.41)	**2.03**	(1.03–3.98)	**1.54**	(1.00–2.35)	2.49*	(1.42–4.36)
**Household income**								
≤60% of MEI	Reference		Reference		Reference		Reference	
60–100% of MEI	0.99	(0.65–1.51)	1.20	(0.74–1.96)	1.27	(0.92–1.77)	1.35	(0.90–2.03)
>100% of MEI	0.87	(0.56–1.37)	0.99	(0.59–1.67)	1.08	(0.76–1.53)	1.45	(0.96–2.20)

Bold numbers = significant at p < 0.05 *p < 0.01 **p < 0.0001.

MEI = median equivalence income.

Female gender significantly predicted homeopathy use (OR = 1.48) and overall CAM use (OR = 1.49). Girls were more likely to be ‘herbal drug users’ as well, but the ORs were not significant for this CAM modality.

Compared with Munich (urban area), the participants from Wesel (rural area) used fewer homeopathic drugs (OR = 0.62) and were less likely to be ‘overall CAM users’ (OR = 0.75). Children from Wesel also consulted CAM providers less (OR = 0.57).

Higher education has a positive effect on CAM use. With the lowest maternal education level as a reference, children whose mother had a university degree showed significantly higher ORs for herbal drug use (OR = 2.03), and consultation with CAM providers (OR = 2.49), but no significant association with educational status was found for homeopathy use.

The equivalence income had no significant impact on any category of medicinal CAM use. However, children from poor households tend to consult CAM providers less. Compared with the lowest income class (up to 60% of MEI), children of parents with 60–100% of MEI visited more CAM providers (OR = 1.35; p = 0.15), and children of parents from the highest income class (more than 100% of MEI) had the highest ORs for consultation with a CAM provider (OR = 1.45; p = 0.08).

### Expenditures on medicinal CAM and CAM providers

Prices were traceable via PZNs for 300 (46.7%) of the 643 reported CAM. The prices of a further 324 CAM were conservatively estimated. The remaining 19 drug entries were not considered for cost analysis as information content was too poor to estimate a price.

The mean price of a homeopathic drug was €10.14 (range: €3.70–€116.69), and the average price for herbal drugs amounted to €13.72 (range: €1.99–€94.45). Looking at all medicinal CAM, the mean cost of one drug was €12.56, ranging between €1.02 and €116.69.

Within a period of 4 weeks, a ‘homeopathy user’ utilized on average homeopathic drugs worth €15.28 (range: €3.70–€124.54). The respective figures for ‘herbal drug users’ were €16.02 (range: €1.99–€94.45) and €18.72 (range: €1.02–€181.22) for ‘overall CAM users’.

A total of 215 ‘CAM provider users’ reported expenditures for consultation with a CAM provider during the previous 12 months. The mean expenditure was €214 (range: €5–€1600).

### Comparison of the results with the results from the 10-year follow-up

Children from the 15-year follow-up (= GINI-15) of the GINIplus birth cohort used significantly less medicinal CAM than those from the 10-year follow-up (= GINI-10). The 4-week prevalence for homeopathy use was more than halved, and herbal drug use dropped by more than a third. In sum, the prevalence of overall medicinal CAM use decreased from 22.3% (GINI-10) to 13.9% (GINI-15), whereas the prevalence of conventional drug use increased from 30.6% to 34.1%.

**Figure 2:**
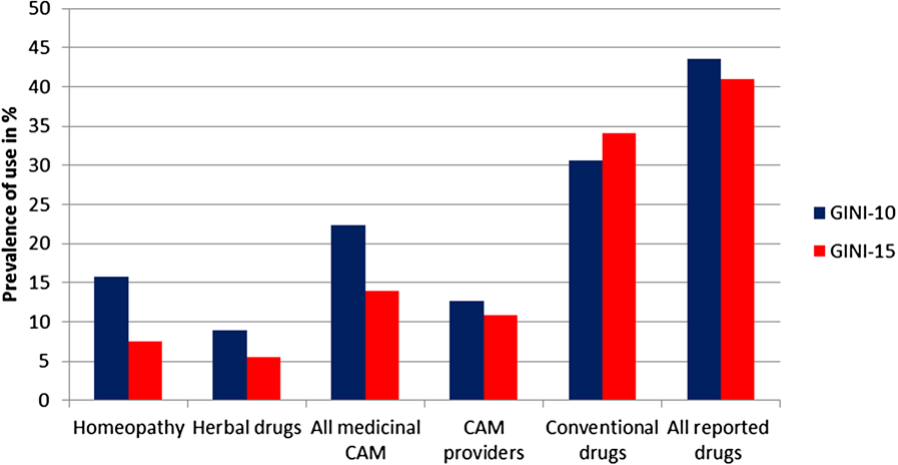
**Comparison of prevalence rates (10-year follow-up (GINI-10) vs. 15-year follow-up (GINI-15)).**

**Figure 3:**
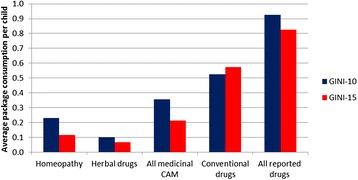
**Average consumption of drug packages (per child in each cohort) in GINI-10 (n = 2065) and GINI-15 (n = 3013).**

**Figure 4:**
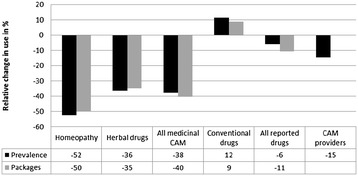
**Relative change in pediatric CAM use (prevalence of use and average consumption of drug packages) in GINI-15 compared with GINI-10.**

The decline in consultations with CAM providers was lower compared with medicinal CAM use. However, the 1-year prevalence fell from 12.6% (GINI-10) to 10.8% (GINI-15). Altogether, the prevalence of overall drug use (conventional + non-conventional drugs put together) did not change significantly. The prevalence rates from both follow-ups and the mean package consumption per child with respect to the various CAM modalities are shown in Figures 2 and 3. Figure 4 visualizes the relative change in CAM utilized, comparing GINI-10 with GINI-15. The findings for predicting factors were in line with the results from the 10-year follow-up.

## Discussion

The present results imply that CAM use in Germany is considerable among 15-year-old children and confirm the popularity of CAM among German children found by other studies [5,6,9,17-19]. A birth cohort study from 2007 [5] analyzing homeopathy use and consultation with a non-medical health practitioner (‘Heilpraktiker’) in 2-year-old children found a 1-year prevalence for homeopathy use of 27.7%. Furthermore, 4.5% of the parents had consulted a ‘Heilpraktiker’ within the last 6 months for their child’s disorder, which is well in line with our findings for GINI-15 (4.8%, 1-year prevalence) and GINI-10 (6.2%, 1-year prevalence). Another study reporting a lifetime prevalence of 85.5% for herbal drug use [9] also included lifetime use of herbal teas such as chamomile or fennel, presumably explaining the very high utilization compared with our findings.

A recent publication on herbal drug use (based on data collected between 2003 and 2006) in a sample of German children aged between 0 and 17 years [18] found a 7-day prevalence of 5.8%. The same data source yielded a 7-day prevalence for homeopathy use of 4.6% [17]. Both results were close to the findings of the present study for homeopathy and herbal drug use, considering the shorter recall period (7 days vs. 4 weeks) which may explain the slightly lower prevalence rates compared with ours. Further results from studies conducted in children with chronic conditions may have yielded higher prevalence rates than in the respective general population and were therefore not considered for comparison with our results.

The children’s financial background seems to have only a weak impact on CAM use in Germany, but education significantly predicted the use of several CAM modalities. Children from the level with the lowest maternal education showed the lowest prevalence of herbal drug use, overall CAM use, and consultation with CAM providers, whereas there was hardly any difference between the three higher education levels. It may be assumed that CAM use is also a matter of health literacy, and children or their mothers from the lowest education stratum are less able or less motivated to inform themselves about health issues such as alternative therapy approaches. Nevertheless, this positive association of drug use with a higher maternal education level was also true for ‘conventional drugs’.

Interestingly, the prevalence rates for medicinal CAM use were substantially lower than the respective figures from the 10-year follow-up in a similar German birth cohort. In contrast to our results, most of the studies reporting a significant association of CAM use with the children’s age found higher prevalence rates in older children [3]. Only three studies (two were German studies) found decreasing prevalence rates with older age [17,18,20]. The fall in CAM use among 15-year-old children (compared with the children from GINI-10) cannot be explained by lower drug utilization in general, as the consumption of ‘conventional drugs’ in GINI-15 did not decrease at the same time. Another German study [21] (using data collected between 2003 and 2006) showed that the prevalence rate for drug utilization in general is not lower in 15-year-old children than in 10-year-old children (children aged between 14 and 17 years were compared with children aged between 7 and 10 years).

Owing to German health legislation from 2004, statutory health insurance covers costs for CAM only in exceptional cases for patients older than 12 years. Therefore, it can reasonably be assumed that most of the medicinal CAM utilized by the children in the present cohort was bought over the counter without medical prescription or a physician’s knowledge.

In contrast to the 15-year follow-up, statutory health insurance would normally cover most of the medicinal CAM prescribed by a physician for the 10-year-old children. De facto, 29.3% of homeopathy, 35.6% of herbal drugs, and 31.3% of overall CAM were prescribed by physicians for the 10-year-old children in GINI-10 (prescription status was assessed in GINI-10 only). A German population-based study also found a reverse association of herbal drug use with children’s age [18]. The authors’ hypothesis is in line with our supposition that this correlation may be due to the possibility of getting expenditure on CAM reimbursed from statutory health insurance. Children may use less CAM if it has to be paid for out of pocket, regardless of their financial background. Consultations with CAM providers were not affected by the reimbursement cuts in the 2004 health act. This may explain the moderate decline in CAM provider visits compared with the substantial fall in medicinal CAM use.

Two longitudinal studies analyzing pediatric CAM use [22,23] found increasing or almost stable prevalence rates over time. A Norwegian publication presented a 1-year prevalence of 8.7% for visits to CAM practitioners among adolescents (17–19 years), an increase of 26% compared with the same group surveyed 4 years before. Another longitudinal study conducted in the UK found only a small variation in homeopathy use, with the same prevalence of 8.0% at the first and last follow-up (at the age of 18 months and 103 months respectively; variable underlying recall periods between 1 and 1.5 years), while the results from the other follow-ups ranged between 5.4% and 6.6%. These results obtained from studies with a longitudinal design further support our hypothesis that the decrease in CAM use in GINI-15 compared with GINI-10 may result from German restrictions (for children older than 12 years) concerning reimbursement for CAM.

This study has strengths and limitations as well. The various CAM modalities were strictly classified and carefully extracted by a pharmacist. Owing to the almost even distribution of data collection over winter, spring, summer, and autumn, the seasonal impact on drug utilization was minimized. The comparably short recall period of 4 weeks presumably reduced recall bias. To our knowledge, only a very few other studies have performed a longitudinal comparison of CAM use over time for cohorts comparable in size and socioeconomic variables [22,23].

Compared with the German mean, the higher education and income levels were overrepresented in the present cohort, because of the disproportionate number of dropouts from the lower socioeconomic levels since the start of the study. Additionally, it must be considered that 15-year-old children may begin to make their own decisions concerning their (self-) medication, and CAM use may also have been influenced by the children’s educational level, which was not assessed in this study. Furthermore, we were unable to rule out non-response bias, as 49.7% of the children recruited at the beginning of the study did not participate in the 15-year follow-up.

With regard to children who consulted a CAM provider, it must be considered that a homeopath may be a conventional physician who uses the term ‘homeopath’ as an additional title. However, a sensitivity analysis that excluded a ‘homeopath’ from the definition as a ‘CAM provider’ yielded no substantial differences with regard to the predictors of consultation with ‘non-conventional’ health providers. The questionnaire for the 15-year follow-up did not explicitly assess whether the reported drugs were prescribed/recommended by a physician or bought on the children’s/parents’ own initiative. Moreover, no information was available on the proportion of privately insured participants among all participating children (with regard to reimbursement for CAM, private health insurance companies may have fewer restrictions than statutory health insurance companies). Therefore, we cannot determine exactly how much of the decrease in CAM use (GINI-15 vs. GINI-10) can be attributed to fewer CAM prescriptions from physicians. Nevertheless, the proportion of over-the-counter drugs (such as medicinal CAM) among all prescribed drugs is estimated to be 17% [24]. The aforementioned figure may be somewhat lower in the present cohort, since the figure refers to the whole German population including children younger than 12 years. Due to potentially different definitions of CAM, the comparability of our results with other international findings may be limited with regard to the predictors and the prevalence of overall CAM use.

Prices for over-the-counter drugs are freely calculable in Germany. The present analysis of expenditures on CAM is based on rough price estimations. Owing to competition, pharmacies may offer CAM at prices lower than those listed in the official price list ‘Lauer’. On the other hand, prices for drugs without available PZNs may have been underestimated by conservative assumptions. Nevertheless, we found no other German studies on pediatric CAM use tracking or estimating prices for the reported remedies utilized.

## Conclusions

Health insurance contributions are mainly generated by the insured persons. Therefore, it may be appropriate that the use of these financial resources should also adequately reflect the obviously existing wish of a noticeable percentage of the German population to integrate CAM into the treatment of their disorders. People with minor ailments may (subjectively) experience a benefit from the use of harmless CAM. At the same time, patients with severe conditions should be aware that CAM is not a suitable substitute for conventional medicine.

The 2004 German health act removed nearly all over-the-counter drugs from the list of reimbursable drugs for children older than 12 years. This may have contributed to the decrease in medicinal CAM use in children from the GINI-15 cohort compared with those from GINI-10, but other reasons such as a possibly lower acceptance of CAM among adolescents (compared with younger children) may have contributed to the drop in CAM use as well. Since 2012 [25], German statutory health insurance companies have again had the possibility to reimburse the costs of over-the-counter drugs (including medicinal CAM such as homeopathy, herbal drugs, etc.). Nevertheless, still many health insurance companies do not cover expenditures on CAM or limit the coverage to a fixed yearly amount [26]. For health insurers, it might be valuable information if reimbursement of CAM influences the decision of insured persons to choose a specific health insurance company.

Future studies assessing exactly how many medicinal CAM are prescribed by physicians may support policy makers and health care managers in their further decision-making process concerning the inclusion of CAM in the list of reimbursable therapy approaches.
